# Hyperglycemia Does Not Affect Iron Mediated Toxicity of Cultured Endothelial and Renal Tubular Epithelial Cells: Influence of L-Carnosine

**DOI:** 10.1155/2016/8710432

**Published:** 2015-12-14

**Authors:** Shiqi Zhang, Emmanouil Ntasis, Sarah Kabtni, Jaap van den Born, Gerjan Navis, Stephan J. L. Bakker, Bernhard K. Krämer, Benito A. Yard, Sibylle J. Hauske

**Affiliations:** ^1^Vth Department of Medicine (Nephrology/Endocrinology/Rheumatology), University Medical Center Mannheim, University of Heidelberg, 68167 Mannheim, Germany; ^2^Department of Endocrinology, The First Affiliated Hospital of Anhui Medical University, Hefei 230022, China; ^3^Department of Cardiology, Pulmonology, Intensive Care and Vascular Medicine, Medical Faculty, RWTH Aachen University, 52074 Aachen, Germany; ^4^Department of Nephrology, University Medical Center Groningen, University of Groningen, 9713 GZ Groningen, Netherlands

## Abstract

Iron has been suggested to affect the clinical course of type 2 diabetes (T2DM) as accompanying increased intracellular iron accumulation may provide an alternative source for reactive oxygen species (ROS). Although carnosine has proven its therapeutic efficacy in rodent models of T2DM, little is known about its efficacy to protect cells from iron toxicity. We sought to assess if high glucose (HG) exposure makes cultured human umbilical vein endothelial cells (HUVECs) and renal proximal tubular epithelial cells (PTECs) more susceptible to metal induced toxicity and if this is ameliorated by L-carnosine. HUVECs and PTECs, cultured under normal glucose (5 mM, NG) or HG (30 mM), were challenged for 24 h with FeCl_3_. Cell viability was not impaired under HG conditions nor did HG increase susceptibility to FeCl_3_. HG did not change the expression of divalent metal transporter 1 (DMT1), ferroportin (IREG), and transferrin receptor protein 1 (TFRC). Irrespective of glucose concentrations L-carnosine prevented toxicity in a dose-dependent manner, only if it was present during the FeCl_3_ challenge. Hence our study indicates that iron induced cytotoxicity is not enhanced under HG conditions. L-Carnosine displayed a strong protective effect, most likely by chelation of iron mediated toxicity.

## 1. Introduction

High body iron levels are associated with increased levels of oxidative stress that may elevate the risk of T2DM [1–7]. Indeed, epidemiological studies have indicated a positive association between high body iron stores and the risk of T2DM and of other insulin resistant states such as metabolic syndrome, gestational diabetes, and polycystic ovarian syndrome [8–11]. High ferritin levels in T2DM are closely related to the development of diabetic vascular complications, possibly through the interaction with vascular endothelial growth factor (VEGF) [12] or through endothelial dysfunction [13]. It has also been suggested that an increased transferrin excretion in diabetic patients with microalbuminuria may contribute to tubulointerstitial injuries [14] as a consequence of transferrin reabsorption by PTECs. This in turn increases intracellular iron concentrations leading to oxidative damage of the tubular cells [7]. The relationship between iron metabolism and T2DM seems to be bidirectional since iron affects glucose metabolism via its deleterious effect on pancreatic *β*-cells, and glucose metabolism impinges on several iron metabolic pathways [4]. However, studies performed in the Belgrade rat, which carries a mutation in the iron transporter DMT1, demonstrate that these rats displayed normal glycemic control and insulin signaling and secretion despite high levels of nonheme iron [15].

It is noteworthy to mention that a number of structures arising secondary to protein glycation might bind transition metals such as iron and copper. The bound metal retains redox active and participates in catalytic oxidation reactions [16]. Iron depletion has been demonstrated to be beneficial in coronary artery responses, endothelial dysfunction, insulin secretion, insulin action, and metabolic control in T2DM.

Carnosine has antioxidant properties and is efficient in the treatment of chemically induced inflammatory lesions in animals [17, 18], fatty acid mediated lipid peroxidation [19], and numerous disease models in which oxidative stress plays a pivotal role [20, 21]. Also in diabetic models oral carnosine supplementation has proven to ameliorate apoptosis of glomerular cells [22], to prevent alterations in carnosine metabolism [23], to improve hyperglycemia [24], and to ameliorate dyslipidemia [25]. These changes are associated with improved renal function, albeit that renal pathology in such models is mostly relatively minor. The mechanism by which carnosine exerts its beneficial effects is multifactorial including chelation of transition metal ions [26, 27], inhibition of advanced glycation end products (AGEs), and inhibition of advanced lipoxidation end products (ALEs) [28].

In keeping with the protective effect of carnosine in diabetic models and the relevance of increased iron stores in T2DM patients, we sought to address if high glucose exposure makes cultured human endothelial cells (ECs) and PTECs more susceptible to iron toxicity and if this is mitigated in the presence of carnosine.

## 2. Methods and Materials

### 2.1. Cell Culture

HUVECs were isolated from fresh human umbilical cords. The cells were cultured on 1% gelatin (Fluka, Neu-Ulm, Germany) coated culture flasks at 37°C and 5% CO_2_ in endothelial cell growth medium (Provitro, Berlin, Germany) containing 50 ng/mL amphotericin B together with 50 *μ*g/mL gentamicin and 2% fetal calf serum (FCS). PTECs were isolated from human kidney biopsy specimen and cultured in DMEM/F-12 medium (DMEM/F-12, GlutaMAX, Invitrogen, Karlsruhe, Germany) supplemented with 36 ng/mL hydrocortisone, 5 *μ*g/mL transferrin, 5 ng/mL sodium selenite, 4 pg/mL triiodo-L-thyronine, 5 *μ*g/mL insulin, and 10 ng/mL EGF at 37°C and 8% CO_2_. The cells were grown on a 0.01% collagen (Sigma, Munich, Germany) coating to which FCS was added. All experiments involving HUVECs and PTECs were performed in passages 3 to 6.

### 2.2. Cell Viability Assay

HUVECs or PTECs were seeded in 96-well plates and cultured for 2 consecutive days under normal glucose (5 mM D-glucose) or high glucose (30 mM D-glucose) conditions. Hereafter the cells were challenged for 24 h with different concentrations of FeCl_3_ (Sigma, Munich, Germany) or ZnCl_2_ (Sigma, Munich, Germany) either in the presence or in the absence of L-carnosine (Sigma, Munich, Germany). In some experiments, the cells were pretreated for 2 days with L-carnosine and subsequently challenged on day 3 with FeCl_3_ in the absence of L-carnosine. Hereafter cytotoxicity was assessed by MTT assay as described in [29]. Cell viability was calculated as percentage relative to cells that were not challenged with FeCl_3_. In each assay and for each condition at least 6 replicates were used. LC_50_ curves were based on at least 4 independent experiments.

### 2.3. TUNEL Assay

DNA damage of HUVECs and PTECs was assessed after treatment by TUNEL assay according to the manufacture's protocol using in situ cell death detection kit fluorescein (Roche, Mannheim, Germany). The excitation wavelength of 488 nm was applied for fluorescence microscope, and the detection wavelength was FITC Green (515–565 nm).

### 2.4. RNA Isolation and qPCR

Total RNA from HUVECs or PTECs was extracted and cDNA was synthesized as described in [30]. qPCR was performed using TaqMan universal PCR master mix (Applied Biosystems, Darmstadt, Germany) on an ABI-Prism 7700 detection system. The TaqMan assays were performed for DMT1 (material number Hs00167206_m1), TFRC (material number Hs00951083_m1), and GAPDH (material number Hs02758991_g1) for normalization.

### 2.5. FACS Analysis

The expression of the TFRC on HUVECs and PTECs was assessed by indirect immune-fluorescence staining and FACS analysis. To this end, cells were incubated for 30 min at 4°C with monoclonal antibodies directed against TFRC (Abcam, Cambridge, UK). Hereafter the cells were thoroughly washed and the 2nd antibodies were conjugated to FITC. The cells were washed twice to remove unbound antibodies and were finally resuspended in 300 *μ*L of Cell Wash (BD Biosciences). Analysis was performed on a FACS Calibur flow cytometer (BD Biosciences) and data were analyzed using WinMDI version 2.8 software.

### 2.6. Western Blot

For detection of DMT1 or carnosinase-1 (CN-1) expressed in HUVECs and PTECs, gel electrophoresis and western blotting were performed. All samples (20 *μ*g of total protein) were 1 : 1 diluted in Laemmli buffer (Bio-Rad, Munich, Germany) and boiled for 5 min before loading on a SDS-PAGE gel. After electrophoresis proteins were transferred to PVDF membranes (Roche, Mannheim, Germany) by semidry blotting as described before [31]. Anti-DMT1 (Abcam, Cambridge, UK) or anti-CNDP1 (Sigma, Munich, Germany) was used as the first antibody afterwards.

### 2.7. Carnosinase Activity

Carnosine was incubated with 300 *μ*M FeCl_3_ for 30 min prior to the initiation of the assay.* CNDP1* recombinant human protein (2 ng/*μ*L) (Life Technology, Darmstadt, Germany) was hereafter added to either carnosine or carnosine-FeCl_3_ mix. Carnosinase activity was afterwards determined according to the method described by Teufel et al. [32].

### 2.8. Lentivirus Transduction

CN-1 cDNA was constructed as described before [24] from IMAGE clone accession number BX094414 and then cloned into HIV based lentivirus vector pPM337. Viruses were produced in HEK293 cells using pCMV891 and pMD.G plasmids. Supernatants of HEK293 cells were collected and concentrated as virus solution. For transduction, HUVECs were incubated in 1 : 100 diluted virus solutions for 48 hours hereafter. Cell lysates were obtained by a freeze-and-thaw cycle in liquid nitrogen. Western blotting of 20 *μ*g total protein from HUVECs supernatant and cell lysates was performed to confirm CN-1 expression. Transduced and wild type HUVECs were challenged with iron in the presence or absence of carnosine.

### 2.9. Statistics

Data between two groups were evaluated by *t*-test, and differences for multiple groups were assessed by one-way ANOVA. Significance was defined according to a *p* < 0.05.

## 3. Results

### 3.1. Susceptibility of Endothelial and Tubular Epithelial Cells to Transition Metals

**Figure 1:**
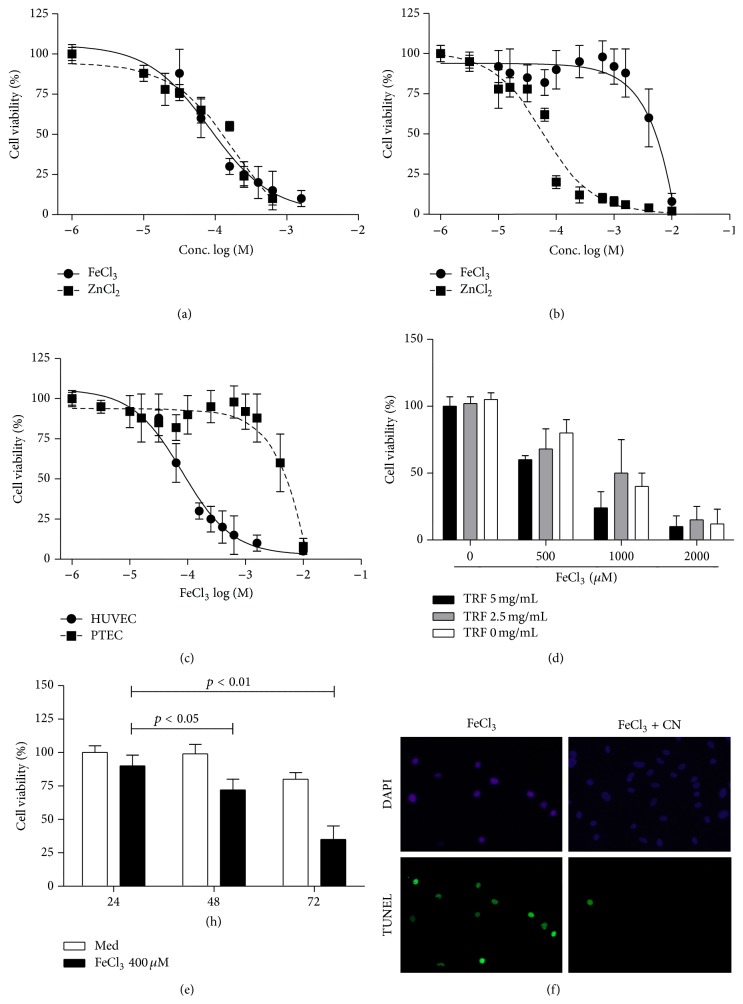
Iron and zinc mediated toxicity in HUVECs (a) and PTECs (b). PTECs were more tolerant to iron toxicity as compared to HUVECs (c). Iron mediated toxicity was not largely influenced by transferrin (TRF) (d). Although PTECs were less susceptible to iron mediated toxicity, toxicity increased upon longer exposure (e). Iron toxicity was associated with an increased number of TUNEL positive cells, which was significantly abrogated by L-carnosine (f).

To assess susceptibility of HUVECs and PTECs to transition metal mediated toxicity cells were challenged for 24 h with different concentrations of FeCl_3_ or ZnCl_2_. While HUVECs were equally susceptible to FeCl_3_ or ZnCl_2_ (LC_50_: ~0.1 mM for both transition metals), PTECs were more susceptible to ZnCl_2_ (LC_50_: ~0.1 mM) as compared to FeCl_3_ (LC_50_: ~5 mM). Susceptibility to FeCl_3_ was significantly higher in HUVECs than in PTECs (Figures 1(a)–1(c)). Since HUVECs medium contains 2% FCS while PTECs are cultured in serum-free culture medium supplemented with transferrin, we tested if this might explain the difference in susceptibility between HUVECs and PTECs. Even if HUVECs were challenged in PTECs medium in the absence of transferrin the cells were highly susceptible to FeCl_3_ and no increase in susceptibility was observed with increasing transferrin concentrations (Figure 1(d)). PTECs became also susceptible to lower concentration of FeCl_3_ (400 *μ*M) if they were challenged over a longer period of time (Figure 1(e)). Transition metal mediated toxicity was associated with DNA damage as evidenced by an increased number of TUNEL positive cells (Figure 1(f)).

### 3.2. Carnosine Prevents Iron Mediated Toxicity Regardless of the Presence of High Glucose

**Figure 2:**
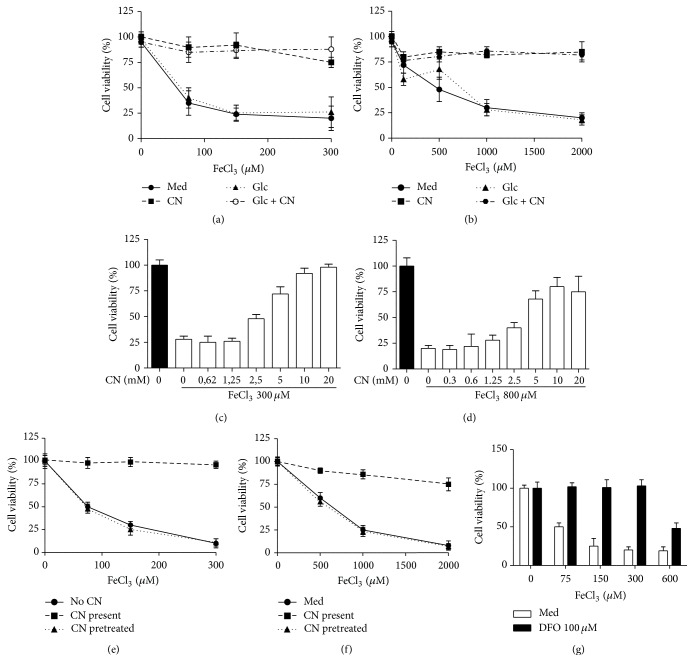
Influence of high glucose and carnosine on iron mediated toxicity in HUVECs (a, c, and e) and PTECs (b, d, and f). High glucose concentration neither increased cell susceptibility to iron nor disrupted the protective effect of carnosine on iron toxicity in HUVECs (a) and PTECs (b). The protective effect of carnosine showed a clear dose-dependent relation in both HUVECs (c) and PTECs (d). Carnosine was only protective when presented during iron challenge but not when cells were pretreated (e and f). Like carnosine also the iron chelator DFO prevented iron mediated toxicity in HUVEC when iron concentration was less than 600 *μ*M (g). Med: medium, CN: carnosine, Glc: high glucose, and Glc + CN: high glucose + carnosine.

**Figure 3:**
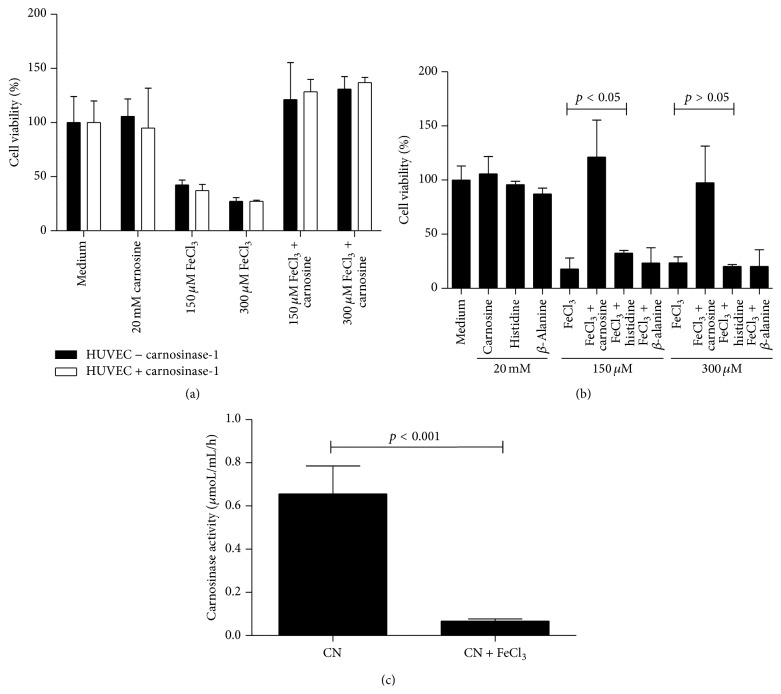
Ectopic expression of the human serum carnosinase-1 in HUVECs did not abrogate the protective effect of carnosine (a). The ability of the constitutive amino acids of carnosine, that is, *β*-alanine and L-histidine, to protect HUVECs against iron mediated toxicity was tested (b). Although L-histidine was slightly protective when iron concentration was 150 *μ*M, protection was clearly less compared to L-carnosine. No protection was afforded by *β*-alanine. Recombinant carnosinase activity was attenuated by the presence of 300 *μ*M FeCl_3_ (c). CN: carnosine.

We next assessed if HG conditions make the cells more vulnerable to FeCl_3_ mediated toxicity. Cell viability of HUVECs and PTECs was not impaired under HG conditions (3 days of culture) nor was HG associated with increased susceptibility to FeCl_3_. When the cells were challenged in the presence of carnosine, toxicity was strongly abrogated under NG and HG conditions (Figures 2(a) and 3(b)). For both HUVECs and PTECs the protective effect of carnosine revealed a dose-dependent relation (Figures 2(c) and 2(d)). Protection only occurred when carnosine was present during the FeCl_3_ challenge but not when carnosine was used as pretreatment only (Figures 2(e) and 2(f)). Similar to carnosine also deferoxamine (DFO) was able to protect HUVECs (Figure 2(g)) and PTECs (data not shown) against iron mediated toxicity, albeit that at higher concentrations of FeCl_3_ the protection by 100 *μ*M of DFO started to wean off. Higher concentrations of DFO were not used as these concentrations negatively affected cell viability, independent of FeCl_3_.

### 3.3. Ectopic CN-1 Expression in HUVECs Does Not Abrogate Protection by Carnosine

To assess if aberrant overexpression of serum carnosinase would mitigate protection against FeCl_3_ mediated toxicity, HUVECs were transduced with* CNDP1* cDNA by lentivirus. CN-1 expression was detected in both cell lysates and supernatants of* CNDP1* transduced HUVECs but not in wild type HUVECs (data not shown). Susceptibility to FeCl_3_ was not changed upon* CNDP1* transduction nor was the protective effect of carnosine abrogated (Figure 3(a)). To exclude that protection by carnosine was at large mediated via its constituent amino acids we performed similar experiments with *β*-alanine or L-histidine. Although L-histidine showed a minimal but significant level of protection when iron concentration was at 150 *μ*M, protection was by far not comparable to that of carnosine (Figure 3(b)). Nevertheless, the minor protective effect of L-histidine was diminished when iron concentration was at 300 *μ*M. Carnosinase protein and activity were found in both the cell lysate and supernatants of* CNDP1* transduced HUVECs but were significantly lower as compared to human serum. We also tested whether carnosine-iron complex would be resistant to the hydrolysis of carnosinase. In this setting, carnosine was incubated with 300 *μ*M FeCl_3_ for 30 min prior to the initiation of carnosinase activity measurement. Indeed carnosinase activity was hardly detectable when iron was added (Figure 3(c)), suggesting either a higher resistance of iron complexed carnosine to hydrolysis by serum carnosinase or direct inhibition of serum carnosinase by iron.

### 3.4. Influence of Iron and Carnosine on Iron Transporters

**Figure 4:**
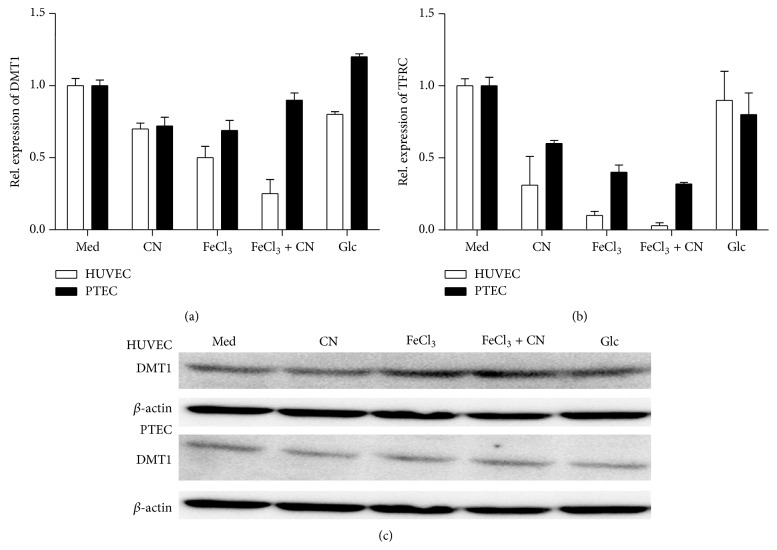
Influence of glucose and L-carnosine on DMT1 (a) and TFRC (b) mRNA expression. HUVECs (open bars) and PTECs (filled bars) were cultured for 24 h in the presence of 20 mM L-carnosine (CN), 60 *μ*M of FeCl_3_, or both combinations. In addition cells were cultured for 3 days in HG medium (Glc). Total RNA was isolated and DMT1 (a) and TFRC (b) mRNA expression were assessed by qPCR. In (c) protein expression of DMT1 in HUVECs and PTECs is shown.

The expression of two major iron transporters, that is, DMT1 and TFRC, was investigated at mRNA and protein level in both HUVECs and PTECs. To avoid toxicity, HUVECs and PTECs were cultured for 24 h in the presence of low iron concentration (60 *μ*M). While in HUVECs DMT1 mRNA was strongly downregulated by iron and carnosine, in PTECs the effect was small (Figure 4(a)). TFRC mRNA expression was downregulated in both HUVECs and PTECs by iron and carnosine (Figure 4(b)). Although downregulation of DMT1 mRNA was observed in the presence of iron and carnosine this was not reflected by a decrease in DMT1 protein expression (Figure 4(c)). In HUVECs TFRC protein expression was slightly decreased by iron, but not by carnosine. In contrast, both iron and carnosine decreased the expression of TFRC on PTECs (Table 1).

## 4. Discussion

A recent trial to assess chelation of transition metals by EDTA, that is, the Trial to Assess Chelation Therapy (TACT) study [33], has provided evidence that intravenous chelation therapy may reduce the risk of mortality and vascular events in diabetics who had previously experienced a myocardial infarction, whereas no benefit was observed in nondiabetics. Also a number of studies have indicated that transition metals, in particularly iron, may have a large impact on the development of diabetes and diabetic complications [34–36]. Since serum carnosinase, encoded by the* CNDP1* gene, has been suggested to be implicated in susceptibility to diabetic nephropathy, the present study was carried out to assess if carnosine, the natural substrate of serum carnosinase, is able to mitigate iron mediated toxicity in endothelial and proximal tubular cells. The main findings of our study are as follows. Firstly, HG does not make endothelial and proximal tubular cells more susceptible to transition metal induced toxicity. HUVECs and PTECs were equally susceptible to Zn^2+^, while for Fe^3+^ PTECs were more resistant as compared to HUVECs. Secondly, carnosine is able to prevent iron mediated toxicity in both cell types in a dose-dependent manner. The presence of serum carnosinase did not abrogate the protective effect of carnosine. Protection by carnosine was likely mediated by chelation of iron as it only occurred when carnosine was present during the iron challenge. Moreover, also deferoxamine was able to prevent iron mediated toxicity. Thirdly, it seems that both iron and carnosine were able to downregulate DMT1 and TFRC mRNA although at the protein level this was only observed for TFRC.

It has been suggested that dietary glycoxidation and lipoxidation products, rich in meat and other animal products, may at large be responsible for the increased risk of T2DM [15, 37, 38]. Glycoxidation and lipoxidation products, which occur after reaction between carbonyl groups on sugars and amine groups on proteins, DNA, and lipoproteins, are abundantly present in diabetic patients and are believed to be instrumental for diabetic complications. It is conceivable that glycoxidative and lipoxidative stress may act in concert with ROS to amplify tissue damage [39, 40]. Our data however do not indicate that HG makes endothelial and proximal tubular cells more susceptible to iron mediated toxicity. It can be argued that this might be a consequence of a relative short exposure to HG; however other studies using similar cells and culture conditions have demonstrated lipid peroxidation already after 48 h [41].

In plasma iron is linked in its ferric form to transferrin. The transferrin bound iron is subsequently taken up by cells through TFRC via an endocytic process. In the endocytic vesicle ferric iron is reduced and transferred to the cytosol via DMT1 [42, 43]. The expression of both TFRC and DMT1 was lower in PTECs as compared to HUVECs which may explain why PTECs were more resistant to iron. Yet it should be underscored that susceptibility of HUVECs to iron was not influenced by the presence of transferrin. This argues against the notion that intracellular iron accumulation via iron-transferrin mediated endocytosis was a major cause of toxicity in our* in vitro* model, albeit that it does not exclude the role of intracellular iron accumulation* per se* for toxicity.

Our data indicate that the protective effect of carnosine likely resides outside the cell. In support of this assumption is the observation that carnosine can only protect against iron mediated toxicity when it is present during the iron challenge. Previously the ferroxidase-like activity of carnosine has been linked to its cytoprotective effect [44]. Since ferroxidase catalyzes the oxidation of Fe^2+^ to Fe^3+^ ferroxidase activity indeed would have the potential to be cell protective as Fe^2+^ may generate damaging hydroxyl radical in the presence of H_2_O_2_. Yet in our study iron toxicity was performed with both Fe^2+^ (data not shown) and Fe^3+^ which in essence were toxic for HUVECs to a similar extent and carnosine was able to prevent toxicity mediated by both iron ions. Nonetheless, since we did not measure ferrous and ferric ions it cannot be excluded that carnosine pushes the equilibrium towards Fe^3+^.

It has also been suggested that complex formation of carnosine with transition metals underlies in part the antioxidative properties of carnosine, albeit that this has been questioned by others [45]. Complex formation has mostly been studied for Zn^2+^ and Cu^2+^ and is mainly attributed to the imidazole moiety within histidine [46, 47]. Like carnosine also DFO was protective, making iron chelation as an alternative explanation for the protective effect of carnosine. Overexpression of serum carnosinase however did not abrogate the protective effect of carnosine, which was explained by the finding that the carnosinase activity was significantly reduced in the presence of iron.

In conclusion our* in vitro* studies clearly demonstrate the ability of carnosine to ameliorate iron mediated toxicity in cultured endothelial and proximal tubular cells. In keeping with the high serum carnosinase concentration in humans, the* in vivo* relevance of our findings needs to be addressed in future studies. The use of carnosine analogues or transition metal ions complexed carnosine that are resistant to hydrolysis by carnosinase may overcome this hurdle. The recent finding that the carnosinase-resistant, D-carnosine, or its bioavailable prodrug D-carnosine octylester has a salutary effect on lipoxidation mediated cellular injury in experimental atherosclerosis and renal disease is promising [48]. Yet it remains to be assessed if such compounds are also able to prevent iron mediated toxicity.

## Figures and Tables

**Table 1:** TFRC expression under different conditions in either HUVECs or PTECs.

TFRC expression %	HUVEC	PTEC
Med	69.2 ± 4.6	30.1 ± 3.7
CN	69.8 ± 3.4	16.5 ± 1.7
Fe (60 *µ*M)	54.0 ± 3.9	23.0 ± 4.7
Fe (60 *µ*M) + CN	70.0 ± 1.0	15.8 ± 3.6

## References

[1] Sampaio A. F. S., Silva M., Dornas W. C. (2014). Iron toxicity mediated by oxidative stress enhances tissue damage in an animal model of diabetes. *BioMetals*.

[2] Murakami K., Okubo H., Sasaki S. (2005). Effect of dietary factors on incidence of type 2 diabetes: a systematic review of cohort studies. *Journal of Nutritional Science and Vitaminology*.

[3] Ikeda Y., Enomoto H., Tajima S. (2013). Dietary iron restriction inhibits progression of diabetic nephropathy in db/db mice. *The American Journal of Physiology—Renal Physiology*.

[4] Fernández-Real J. M., López-Bermejo A., Ricart W. (2002). Cross-talk between iron metabolism and diabetes. *Diabetes*.

[5] Rajpathak S., Ma J., Manson J., Willett W. C., Hu F. B. (2006). Iron intake and the risk of type 2 diabetes in women: a prospective cohort study. *Diabetes Care*.

[6] Luan D. C., Li H., Sui J. L., Zhao Z., Li X., Zhong M. L. (2008). Body iron stores and dietary iron intake in relation to diabetes in adults in North China. *Diabetes Care*.

[7] Martines A. M. F., Masereeuw R., Tjalsma H., Hoenderop J. G., Wetzels J. F. M., Swinkels D. W. (2013). Iron metabolism in the pathogenesis of iron-induced kidney injury. *Nature Reviews Nephrology*.

[8] Ford E. S., Cogswell M. E. (1999). Diabetes and serum ferritin concentration among U.S. adults. *Diabetes Care*.

[9] Zein S., Rachidi S., Hininger-Favier I. (2014). Is oxidative stress induced by iron status associated with gestational diabetes mellitus?. *Journal of Trace Elements in Medicine and Biology*.

[10] Javadian P., Alimohamadi S., Gharedaghi M. H., Hantoushzadeh S. (2014). Gestational diabetes mellitus and iron supplement; Effects on pregnancy outcome. *Acta Medica Iranica*.

[11] Sam A. H., Busbridge M., Amin A. (2013). Hepcidin levels in diabetes mellitus and polycystic ovary syndrome. *Diabetic Medicine*.

[12] Guo L., Jiang F., Tang Y.-T., Si M.-Y., Jiao X.-Y. (2014). The association of serum vascular endothelial growth factor and ferritin in diabetic microvascular disease. *Diabetes Technology & Therapeutics*.

[13] Duffy S. J., Biegelsen E. S., Holbrook M. (2001). Iron chelation improves endothelial function in patients with coronary artery disease. *Circulation*.

[14] Kanauchi M., Akai Y., Hashimoto T. (2002). Transferrinuria in type 2 diabetic patients with early nephropathy and tubulointerstitial injury. *European Journal of Internal Medicine*.

[15] Uribarri J., Cai W., Sandu O., Peppa M., Goldberg T., Vlassara H. (2005). Diet-derived advanced glycation end products are major contributors to the body's AGE pool and induce inflammation in healthy subjects. *Annals of the New York Academy of Sciences*.

[16] Qian M., Liu M., Eaton J. W. (1998). Transition metals bind to glycated proteins forming redox active “glycochelates”: implications for the pathogenesis of certain diabetic complications. *Biochemical and Biophysical Research Communications*.

[17] Yoshikawa T., Yamaguchi T., Yoshida N. (1997). Effect of Z-103 on TNB-induced colitis in rats. *Digestion*.

[18] Watari I., Oka S., Tanaka S. (2013). Effectiveness of polaprezinc for low-dose aspirin-induced small-bowel mucosal injuries as evaluated by capsule endoscopy: a pilot randomized controlled study. *BMC Gastroenterology*.

[19] Rogers C., Davis B., Neufer P. D., Murphy M. P., Anderson E. J., Robidoux J. (2014). A transient increase in lipid peroxidation primes preadipocytes for delayed mitochondrial inner membrane permeabilization and ATP depletion during prolonged exposure to fatty acids. *Free Radical Biology and Medicine*.

[20] Zhang Z. Y., Sun B., Yang M., Li D., Fang J., Zhang S. (2015). Carnosine attenuates early brain injury through its antioxidative and anti-apoptotic effects in a rat experimental subarachnoid hemorrhage model. *Cellular and Molecular Neurobiology*.

[21] Giriş M., Doğru-Abbasoğlu S., Kumral A., Olgaç V., Koçak-Toker N., Uysal M. (2014). Effect of carnosine alone or combined with *α*-tocopherol on hepatic steatosis and oxidative stress in fructose-induced insulin-resistant rats. *Journal of Physiology and Biochemistry*.

[22] Riedl E., Pfister F., Braunagel M. (2011). Carnosine prevents apoptosis of glomerular cells and podocyte loss in stz diabetic rats. *Cellular Physiology and Biochemistry*.

[23] Peters V., Schmitt C. P., Zschocke J., Gross M.-L., Brismar K., Forsberg E. (2012). Carnosine treatment largely prevents alterations of renal carnosine metabolism in diabetic mice. *Amino Acids*.

[24] Sauerhöfer S., Yuan G., Braun G. S. (2007). L-carnosine, a substrate of carnosinase-1, influences glucose metabolism. *Diabetes*.

[25] Aldini G., Orioli M., Rossoni G. (2011). The carbonyl scavenger carnosine ameliorates dyslipidaemia and renal function in Zucker obese rats. *Journal of Cellular and Molecular Medicine*.

[26] Torreggiani A., Bonora S., Fini G. (2000). Raman and IR spectroscopic investigation of zinc(II)-carnosine complexes. *Biopolymers*.

[27] Torreggiani A., Tamba M., Fini G. (2000). Binding of copper(II) to carnosine: raman and IR spectroscopic study. *Biopolymers*.

[28] Song B. C., Joo N.-S., Aldini G., Yeum K.-J. (2014). Biological functions of histidine-dipeptides and metabolic syndrome. *Nutrition Research and Practice*.

[29] Stamellou E., Storz D., Botov S. (2014). Different design of enzyme-triggered CO-releasing molecules (ET-CORMs) reveals quantitative differences in biological activities in terms of toxicity and inflammation. *Redox Biology*.

[30] Stamellou E., Fontana J., Wedel J. (2014). N-octanoyl dopamine treatment of endothelial cells induces the unfolded protein response and results in hypometabolism and tolerance to hypothermia. *PLoS ONE*.

[31] Adelmann K., Frey D., Riedl E. (2012). Different conformational forms of serum carnosinase detected by a newly developed sandwich ELISA for the measurements of carnosinase concentrations. *Amino Acids*.

[32] Teufel M., Saudek V., Ledig J. P. (2003). Sequence identification and characterization of human carnosinase and a closely related non-specific dipeptidase. *The Journal of Biological Chemistry*.

[33] Escolar E., Lamas G. A., Mark D. B. (2014). The effect of an EDTA-based chelation regimen on patients with diabetes mellitus and prior myocardial infarction in the Trial to Assess Chelation Therapy (TACT). *Circulation: Cardiovascular Quality and Outcomes*.

[34] Chace K. V., Carubelli R., Nordquist R. E. (1991). The role of nonenzymatic glycosylation, transition metals, and free radicals in the formation of collagen aggregates. *Archives of Biochemistry and Biophysics*.

[35] Ou P., Wolff S. P. (1994). Erythrocyte catalase inactivation (H_2_O_2_ production) by ascorbic acid and glucose in the presence of aminotriazole: role of transition metals and relevance to diabetes. *Biochemical Journal*.

[36] Mowri H.-O., Frei B., Keaney J. F. (2000). Glucose enhancement of LDL oxidation is strictly metal ion dependent. *Free Radical Biology and Medicine*.

[37] Feskens E. J. M., Sluik D., Van Woudenbergh G. J. (2013). Meat consumption, diabetes, and its complications. *Current Diabetes Reports*.

[38] Van Dam R. M., Willett W. C., Rimm E. B., Stampfer M. J., Hu F. B. (2002). Dietary fat and meat intake in relation to risk of type 2 diabetes in men. *Diabetes Care*.

[39] Hansen J. B., Moen I. W., Mandrup-Poulsen T. (2014). Iron: the hard player in diabetes pathophysiology. *Acta Physiologica*.

[40] Swaminathan S., Fonseca V. A., Alam M. G., Shah S. V. (2007). The role of iron in diabetes and its complications. *Diabetes Care*.

[41] Park M. H., Han J. S. (2013). Protective effect of padina arborescens extract against high glucose-induced oxidative damage in human umbilical vein endothelial cells. *Preventive Nutrition and Food Science*.

[42] Fleming M. D., Romano M. A., Maureen A. S. U., Garrick L. M., Garrick M. D., Andrews N. C. (1998). Nramp2 is mutated in the anemic Belgrade (b) rat: evidence of a role for Nramp2 in endosomal iron transport. *Proceedings of the National Academy of Sciences of the United States of America*.

[43] Fleming M. D., Trenor C. C., Su M. A. (1997). Microcytic anaemia mice have a mutation in Nramp2, a candidate iron transporter gene. *Nature Genetics*.

[44] Yoshikawa T., Naito Y., Tanigawa T., Yoneta T., Kondo M. (1991). The antioxidant properties of a novel zinc-carnosine chelate compound, N-(3-aminopropionyl)-l-histidinato zinc. *Biochimica et Biophysica Acta*.

[45] Velez S., Nair N. G., Reddy V. P. (2008). Transition metal ion binding studies of carnosine and histidine: biologically relevant antioxidants. *Colloids and Surfaces B: Biointerfaces*.

[46] Boldyrev A. A., Aldini G., Derave W. (2013). Physiology and pathophysiology of carnosine. *Physiological Reviews*.

[47] Hobart L. J., Seibel I., Yeargans G. S., Seidler N. W. (2004). Anti-crosslinking properties of carnosine: significance of histidine. *Life Sciences*.

[48] Menini S., Iacobini C., Ricci C. (2012). D-carnosine octylester attenuates atherosclerosis and renal disease in ApoE null mice fed a Western diet through reduction of carbonyl stress and inflammation. *British Journal of Pharmacology*.

